# Intravenous fosfomycin therapy in critically ill patients infected with colistin-resistant enterobacteriacae

**DOI:** 10.1186/cc14199

**Published:** 2015-03-16

**Authors:** DN Mukherjee, L Agarwal, I Nayyar

**Affiliations:** 1 Woodlands Hospital, Kolkata, India

## Introduction

Carbapenem-resistant enterobacteriacae emerged in recent years as one of the most challenging groups of antibiotic-resistant pathogens. Polymyxins are considered as the last resort for the treatment of infections with carbapenem-resistant Gram-negative bacilli (GNB). Inadequate or extensive use of colistin leads to emergence of colistin resistance in GNB, jeopardizing treatment options in ICUs, potentially increasing mortality and morbidity and necessitating prudent use of alternative antibiotics. Fosfomycin, a phosponic acid derivative which acts primarily by disrupting bacterial cell wall synthesis, is a broad-spectrum antibiotic. Fosfomycin tromethamine is an oral formulation approved for the treatment of uncomplicated urinary tract infection caused by multidrug-resistant (MDR) bacteria. Recently fosfomycin is also available as a sodium/disodium formulation for intravenous use, which is showing promising result against MDR/ potentially drug-resistant pathogens.

## Methods

A total of four colistin-resistant (MIC ≥4) GNB were isolated from ICU patients with nosocomial MDR infections. All four isolates were Klebsiella pneumonia. Among these isolates three were from blood and one from endotracheal aspirate and all four isolates were sensitive to fosfomycin *in vitro*. All of these patients had multiple comorbidities with recent history of colistin exposure. Intravenous fosfomycin sodium (inj Fosmicin; Meiji, Japan) was started as a combination therapy with carbapenem.

## Results

Among the three bacteremic patients, two recovered completely from sepsis as well as the patient with ventilator-associated pneumonia. There was clinical as well as microbiological cure with normalization of sepsis markers. The only one bacteremic patient who died during the course of therapy was later diagnosed to have azoleresistant fungemia as a superinfection.

## Conclusion

Based on the evidence of clinical experience and available studies, intravenous fosfomycin therapy may be considered as the last option for the treatment of MDR GNB infection where there is documented colistin resistance and where there is literally no other choice of antibiotic therapy.
